# The ‘good death’ in Mainland China - A Scoping Review

**DOI:** 10.1016/j.ijnsa.2022.100069

**Published:** 2022-02-12

**Authors:** Cong Fu, Stinne Glasdam

**Affiliations:** a Integrative Health Research, Department of Health Sciences, Faculty of Medicine, Lund University, Margaretavägen 1 B, S- 222 41 Lund, Sweden.; b Integrative Health Research, Department of Health Sciences, Faculty of Medicine, Lund University, Margaretavägen 1 B, S- 222 41 Lund, Sweden.

**Keywords:** The good death, Mainland China, Scoping Review, Chinese philosophy, western philosophy

## Abstract

**Background:**

Since the mid 80’ies, the western palliative care philosophy has influenced the development of palliative care in mainland China. However, it has caused several challenges.

**Objective:**

To explore the understanding of the ‘good death’ among authorities, professionals, patients, and their relatives in end-of-life care settings in mainland China.

**Design:**

Scoping review. The PRISMA-ScR checklist was used. The study is not registered.

**Settings:**

End-of-life care settings, Mainland China

**Participants:**

Authorities, healthcare professionals, adult patients, and general population in mainland China.

**Method:**

Literature searches were performed through Medline, CINAHL, PsycInfo, and Web of Sciences from 2001-2021, last search 21.4.2021. Inclusion criteria were: Empirical research studies investigating ‘good death’ or political documents about ‘good death’, perspectives from authorities, professionals, patients, and/or relatives, and studies following the Declaration of Helsinki. Exclusion criteria were: Literature reviews, languages other than English and Chinese, editorials, letters, comments, and children's death/dying.The analysis consisted of analysing the data including a descriptive numerical summary analysis and a qualitative thematic analysis.

**Results:**

Nineteen articles and two political documents were included. The 19 studies were carried out from 2003-2020, with data collected from 1999 to 2019. The political documents were written in 2012 and 2017, respectively. The thematic analysis resulted in three themes: ‘Medicalisation of death’, ‘Communication about death - a clash between two philosophies’, and ‘Dying and death were socially dependent’. The medicalisation of death meant the understanding of the ‘good death’ primarily focused on physical symptoms and treatments. The good death was understood as painless and symptom-free, where all symptoms could be measured and assessed. Dignity and shared decision-making were connected to the understanding of the ‘good death’. However, the contents of the ‘good death’ varied across the different actors. The understanding of the ‘good death’ in mainland China was a negotiation between Chinese traditional philosophy and contemporary western medicine practice. There was a tension between openness and silence about death, which reflected the importance of death education. The understanding of the ‘good death’ consisted partly of a timely and practical preparation for the death and afterlife, partly of a matter of social and financial issues.

**Conclusions:**

There seemed to be a clash between two different cultures in the understanding of a good death in Mainland China, where western philosophy seemed to rule the political medical actors while traditional Chinese philosophy seemed to rule parts of the population.

What is already known about the topic?

• The awareness and development of palliative care in mainland China lagged behind the western world.
• The idea of the ‘good death’ was poorly explored in mainland China.

What does this paper add?

• In mainland China, the ‘good death’ was understood in between the traditional Chinese philosophy of familism and the western medical individualism.
• In the traditional Chinese tradition, the ‘good death’ depended partly on a timely and practical preparation for the death, passing, and afterlife, partly on social and financial issues.
• Chinese authorities, health organisations, and healthcare professionals supported the western medical understanding of the ‘good death’.

## 1 Introduction

This article focuses on palliative care in mainland China, defined by the World Health Organisation (WHO) as: “... an approach that improves the quality of life of patients and their families facing the problem associated with life-threatening illness, through prevention and relief of suffering by means of early identification and impeccable assessment and treatment of pain and other problems, physical, psychosocial and spiritual.” (World Health Organization 2018). Palliative care is a multidisciplinary approach aiming to address sufferings and symptoms, providing so-called holistic care and support. It is based on patients' and their families’ needs, regardless of the type or stage of disease (World Health Organization 2018). The modern hospice movement and its philosophy of care developed as a critique of the hospitalisation of dying in western societies (Ariès, 1981, Saunders et al., 1961). The hospice philosophy included an idea of the ‘good death’ aiming to help dying people to a good and peaceful death characterised by open awareness, acceptance, and reconciliation with dying (Saunders, 2018). Studies show that even though hospice developed as a critical response to medicine, medicine is central to hospice care (Clark, 2016, Graven and Timm, 2019, Graven et al., 2020, Robinson et al., 2017). The awareness of dying, open communication, acceptance of death, and patient's autonomy in treatment decisions are highlighted as key elements in the ‘good death’ in the western palliative care philosophy (Wilson et al., 2014, Brogaard et al., 2013, McNamara, 2004). Elements of this understanding of the ‘good death’ appear across different cultures and societies. However, studies show that the understanding of the ‘good death’ varies across ethnic groups and societal classes all over the world (Cain and McCleskey, 2019, Cha et al., 2021, Gafaar et al., 2020 Jul 10, Kim, 2019, Meier et al., 2016, Mo et al., 2012, Miyashita et al., 2007, Tayeb et al., 2010, Watanabe et al., 2008 Feb 27).

China is the most populous country with a population of 1,4 billion (United Nations 2021). In 2020, there were approximately 9 million deaths in mainland China (Statista 2021). Mainland China refers to the territories governed by People's Republic China, excluding Hong Kong, Macau, and Taiwan. The introduction of palliative care in mainland China occurred in 1988, but the development of palliative care has been slow (Yan et al., 2020). The availability of hospice and palliative care services are restricted to major cities (Wang et al., 2018). A ten-year-old study shows that 90% of patients with advanced cancer have no access to the palliative care services in China (Li et al., 2011). Mainland China ranked 71st out of 80 worldwide in the Quality of Death Index 2015, lagging behind top-rated countries such as the UK (1st), Belgium (3rd), as well as its Asian counterparts Taiwan (6th), Singapore (12th), Japan (14th) and Hong Kong (22nd) (The Economist Intelligence Unit, 2015). This index reflects the limited accessibility and poor quality of palliative care and the needs for improvement in mainland China (The Economist Intelligence Unit, 2015). Although the urgent need to develop palliative care in mainland China is recognised (Yan et al., 2020, Li et al., 2011), the lack of government support, the cultural misconception towards palliative care, and low awareness of palliative care among healthcare professionals are main barriers in mainland China (Yan et al., 2020, Lu et al., 2018). The philosophy of palliative care is to improve the quality of death and achieve a ‘good death’. Yet what ‘good death’ means to the public, healthcare professionals as well as authorities in mainland China are not fully understood. Therefore, this article aims to explore the understanding of the ‘good death’ among Chinese authorities, healthcare professionals, patients, and their relatives in end-of-life care settings in mainland China.

## 2 Method

A scoping review inspired by (Arksey and O'Malley, 2005) was conducted in April - May 2021. The method of current scoping review will be presented below in five stages: 1) Identifying the research question, 2) identifying relevant studies, 3) study selection, 4) charting the data, and 5) analytical strategy (Arksey and O'Malley, 2005). Studies with a range of study design can be included in a scoping review as such reviews are especially relevant for mapping areas with emerging evidence and a lack of randomised controlled trials (Levac et al., 2010, Munn et al., 2018). A scoping review was relevant for the aim of current study as the literature in this field of ‘mainland Chinese end-of-life care’ was limited and characterised by studies with a wide range of designs and relatively broad research questions. The review was not registered. The article was guided by the PRISMA-ScR checklist (Tricco et al., 2018).

### 2.1 Identifying the research question

What are the components that constitute the ‘good death’ in palliative care settings from patients, family members, healthcare professionals’, and authorities’ perspectives in mainland China?

### 2.2 Identifying relevant studies

A systematic literature search was conducted in three databases: Medline, CINAHL, and PsycInfo. In each of the three selected databases, the search strategy consisted of a building block search carried out according to the populations, exposures, and outcomes (PEO) model (Bettany-Saltikov, 2016); 1) Population: Authorities, healthcare professionals, patients, relatives. 2) Exposure: Palliative care, and 3) Outcome/Theme: Good death, see Table 1. Each block included a variation of relevant search terms. The thesaurus from each database were used to search literature. In addition, free text search terms were used to identify synonyms. Each thesaurus and free text search term in the same block were searched separately and then combined with the Boolean Operator ‘OR’. Finally, the full search string from each block was combined with the Boolean Operator ‘AND’. The search was limited to articles written in English and Chinese and published from 1st January 2001 - 24th April 2021. The full strategy strings were presented in Table 2.

**Table 1:** Population, Exposure and Outcome/theme, PEO

Block 1 (P) – Population	Block 2 (E) - Exposure	Block 3 (O) - Outcome/Theme
Politician* OR Authorit* OR Government OR Public Agency OR Health* administrator* OR	End of life care OR Palliative care OR Terminal care OR Death care OR Hospice care OR Homecare OR home care	Good death OR Dying well OR Successful dying OR Successful death OR Peaceful dying OR Peaceful death OR Attitude* to death OR Attitude* to dying OR straget* for palliative care OR straget* for terminal care OR straget* for dying
Health* professional* OR Nurse* OR Doctor* OR Physician* OR Social worker* OR		
OR Patient* OR client* OR citizen* OR terminal ill person* OR		
Relative* OR Famil* OR Spouse* OR Husband* OR Wife* OR Child* OR son* OR Daughter* OR Social network* OR next of kin*

**Table 2:** The full electronic search strategy for all three databases

Database	Permalink resp. search string
CINAHL	http://ludwig.lub.lu.se/login?url=https://search.ebscohost.com/login.aspx?direct=true&AuthType=ip,uid&db=ccm&bquery=((MH+%26quot%3bOrganizations%2b%26quot%3b)+OR+(MH+%26quot%3bGovernment+Agencies%2b%26quot%3b)+OR+(MH+%26quot%3bNurses%2b%26quot%3b)+OR+(MH+%26quot%3bPhysicians%2b%26quot%3b)+OR+(MH+%26quot%3bSocial+Workers%26quot%3b)+OR+(MH+%26quot%3bHealth+Personnel%2b%26quot%3b)+OR+(MH+%26quot%3bPatients%2b%26quot%3b)+OR+(MH+%26quot%3bCritically+Ill+Patients%26quot%3b)+OR+(MH+%26quot%3bCancer+Patients%26quot%3b)+OR+(MH+%26quot%3bFamily%2b%26quot%3b)+OR+(MH+%26quot%3bSpouses%26quot%3b)+OR+(MH+%26quot%3bSons%26quot%3b)+OR+(MH+%26quot%3bSiblings%26quot%3b)+OR+(MH+%26quot%3bDaughters%26quot%3b)+OR+(MH+%26quot%3bChild%2b%26quot%3b)+OR+(MH+%26quot%3bExtended+Family%2b%26quot%3b)+OR+(MH+%26quot%3bSocial+Networking%2b%26quot%3b)OR+%26quot%3bpolitician%26quot%3b+OR+(health*+AND+administrator*)+OR+(Health*+AND+administration*))+AND+((MH+%26quot%3bPalliative+Care%26quot%3b)+OR+(MH+%26quot%3bHospice+Care%26quot%3b)+OR+(MH+%26quot%3bTerminal+Care%2b%26quot%3b)+OR+(MH+%26quot%3bDeath+Education%26quot%3b)+OR+%26quot%3bend+of+life+care%26quot%3b+OR+(MH+%26quot%3bHome+Health+Care%2b%26quot%3b)+OR+%26quot%3bhomecare%26quot%3b+OR+%26quot%3bterminal+care+unit%26quot%3b+OR+%26quot%3bpalliative+care+unit%26quot%3b)+AND+((MH+%26quot%3bAttitude+to+Death%2b%26quot%3b)+OR+TX%26quot%3bgood+dying%26quot%3b+OR+TX+%26quot%3bgood+death%26quot%3b+OR+TX+%26quot%3bdying+well%26quot%3b+OR+TX+%26quot%3bsuccessful+dying%26quot%3b+OR+TX+%26quot%3bsuccessful+death%26quot%3b+OR+TX+%26quot%3bpeaceful+dying%26quot%3b+OR+TX+%26quot%3bpeaceful+death%26quot%3b+OR+%26quot%3bpalliative+care+strateg*%26quot%3b+OR+%26quot%3bterminal+care+strateg*%26quot%3b+OR+TX+%26quot%3battitude+to+dying%26quot%3b+OR+TX+%26quot%3bquality+of+death%26quot%3b)+AND+(%26quot%3bmainland+china%26quot%3b+OR+(MH+%26quot%3bChina%2b%26quot%3b))&type=1&searchMode=And&site=ehost-live
PsychInfo	http://ludwig.lub.lu.se/login?url=https://search.ebscohost.com/login.aspx?direct=true&AuthType=ip,uid&db=psyh&bquery=(((((((((((DE+%26quot%3bHealth+Care+Administration%26quot%3b+OR+DE+%26quot%3bClinical+Governance%26quot%3b+OR+DE+%26quot%3bHospital+Administration%26quot%3b)+OR+(DE+%26quot%3bGovernment%26quot%3b))+OR+(DE+%26quot%3bPublic+Opinion%26quot%3b))+OR+(DE+%26quot%3bClinicians%26quot%3b))+OR+(DE+%26quot%3bSocial+Workers%26quot%3b+OR+DE+%26quot%3bPsychiatric+Social+Workers%26quot%3b))+OR+(DE+%26quot%3bClient+Attitudes%26quot%3b+OR+DE+%26quot%3bClient+Satisfaction%26quot%3b))+OR+(DE+%26quot%3bTerminally+Ill+Patients%26quot%3b))+OR+(DE+%26quot%3bFamily%26quot%3b+OR+DE+%26quot%3bBiological+Family%26quot%3b+OR+DE+%26quot%3bDual+Careers%26quot%3b+OR+DE+%26quot%3bDysfunctional+Family%26quot%3b+OR+DE+%26quot%3bExtended+Family%26quot%3b+OR+DE+%26quot%3bFamily+Background%26quot%3b+OR+DE+%26quot%3bFamily+History%26quot%3b+OR+DE+%26quot%3bFamily+Members%26quot%3b+OR+DE+%26quot%3bFamily+of+Origin%26quot%3b+OR+DE+%26quot%3bFamily+Relations%26quot%3b+OR+DE+%26quot%3bFamily+Resemblance%26quot%3b+OR+DE+%26quot%3bFamily+Structure%26quot%3b+OR+DE+%26quot%3bFamily+Work+Relationship%26quot%3b+OR+DE+%26quot%3bInterethnic+Family%26quot%3b+OR+DE+%26quot%3bInterracial+Family%26quot%3b+OR+DE+%26quot%3bMilitary+Families%26quot%3b+OR+DE+%26quot%3bNepotism%26quot%3b+OR+DE+%26quot%3bNuclear+Family%26quot%3b+OR+DE+%26quot%3bSchizophrenogenic+Family%26quot%3b+OR+DE+%26quot%3bStepfamily%26quot%3b))+OR+(DE+%26quot%3bSpouses%26quot%3b+OR+DE+%26quot%3bHusbands%26quot%3b+OR+DE+%26quot%3bWives%26quot%3b))+OR+(DE+%26quot%3bPoliticians%26quot%3b))+AND+((DE+%26quot%3bDeath+Attitudes%26quot%3b)+OR+(TX+(%26quot%3bpalliative+care+strategy+%26quot%3bOR+%26quot%3bterminal+care+strategy%26quot%3b+OR+%26quot%3bgood+death%26quot%3b+OR+%26quot%3bpeaceful+dying%26quot%3b+OR+%26quot%3bpeaceful+death%26quot%3b+OR+%26quot%3bdying+well%26quot%3b+OR+%26quot%3bsuccessful+death%26quot%3b+OR+%26quot%3bsuccessful+dying%26quot%3b+OR+%26quot%3bquality+death%26quot%3b+OR+%26quot%3bdignified+death%26quot%3b+OR+%26quot%3bdying+with+dignity%26quot%3b+OR+%26quot%3battitude+to+dying%26quot%3b)))+AND+(((((DE+%26quot%3bDeath+Anxiety%26quot%3b+OR+DE+%26quot%3bPalliative+Care%26quot%3b+OR+DE+%26quot%3bDeath+and+Dying%26quot%3b+OR+DE+%26quot%3bHospice%26quot%3b)+OR+(DE+%26quot%3bDeath+Attitudes%26quot%3b))+OR+(DE+%26quot%3bTerminal+Cancer%26quot%3b))+OR+(DE+%26quot%3bHome+Care%26quot%3b))+OR+(TX+(%26quot%3bquality+of+death+%26quot%3bOR+%26quot%3bterminal+care+units%26quot%3b+OR+%26quot%3bpalliative+care+unit+%26quot%3b+OR+%26quot%3bterminal+care%26quot%3b+OR+%26quot%3b+death+care%26quot%3b+OR+%26quot%3bend+of+life+care%26quot%3b))))+AND+(TX+(%26quot%3bChina%26quot%3b+OR+%26quot%3bmainland+China+%26quot%3b))&type=1&searchMode=And&site=ehost-live
PubMed	"authorship/organization and administration"[MeSH Terms] OR "government"[MeSH Terms] OR "public agency"[All Fields] OR health* administrator* OR Health* administration* OR "nurses"[MeSH Terms] OR "physicians"[MeSH Terms] OR "social workers"[MeSH Terms] OR "healthcare professionals"[All Fields] OR "patients"[MeSH Terms] OR "terminally ill patients"[All Fields] OR "citizen*"[All Fields] OR "family"[MeSH Terms] OR "spouses"[MeSH Terms] OR "child"[MeSH Terms] OR "relatives"[All Fields] OR "next of kin"[All Fields] OR "politician"[Text Word] OR "social networking"[MeSH Terms] AND "palliative care"[MeSH Terms] OR "hospice care"[MeSH Terms] OR "terminal care"[MeSH Terms] OR "death care"[Text Word] OR "end of life care"[Text Word] OR "home care services"[MeSH Terms] OR "home care"[Text Word] OR "homecare"[Text Word] OR "terminal care unit*"[Text Word] OR "palliative care unit*"[Text Word] AND "attitude to death"[MeSH Terms] OR "good death"[Text Word] OR "dying well"[Text Word] OR "successful dying"[Text Word] OR "successful death"[Text Word]OR "peaceful dying"[Text Word] OR "peaceful death"[Text Word] OR "palliative care strateg*"[Text Word] OR "good death "OR " good dying " [Text Word] OR "terminal care strateg*" OR "attitude* to dying"OR "quality of death"[Text Word] AND "mainland china" OR MH "China+"

The 117 identified studies were transferred to COVIDENCE.org for the following screening process. We supplemented the building block with a citation pearl search in the Web of Science citation database of the included articles to find studies outside the three databases. In addition, we included grey literature (Paez, 2017) in the form of materials about strategies of dying and palliative care produced by governments and health organisations. We searched for grey literature at the homepages of the Chinese government, published by Chinese healthcare authorities, and any recognised palliative care organisation/association identified through Global Directory of Palliative Care.

### 2.3 Study selection

Both authors separately screened the studies’ title, abstract and full text using COVIDENCE.org with following criteria: Empirical research studies investigating ‘good death’ or authority documents about ‘good death’, perspectives from authorities, healthcare professionals, patients, and/or relatives, and studies following the ethical principles of the Declaration of Helsinki (World Medical Association 2013). Following articles were excluded: Literature reviews, other languages than English and Chinese, editorials, letters, comments, children's death/dying, studies only conducted in Hong Kong, Macau, and/or Taiwan.

**Fig. 1:**
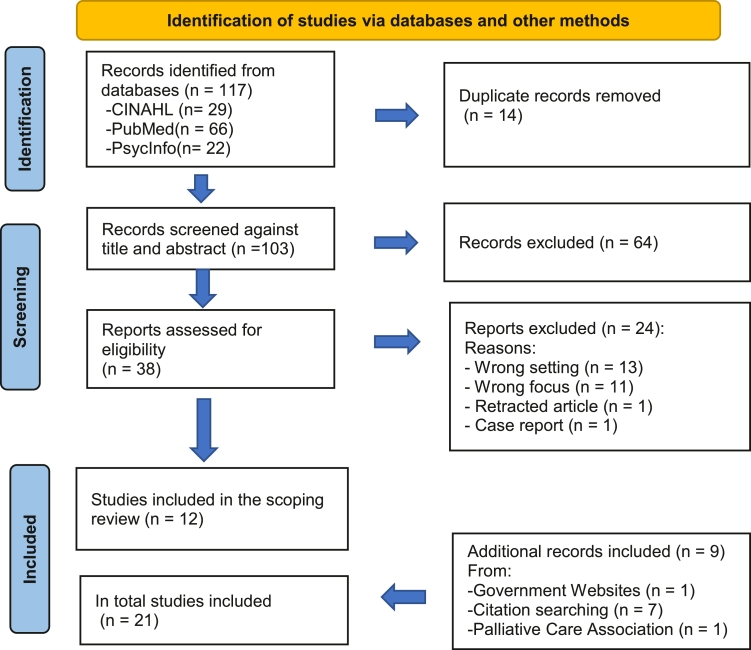
From Page MJ, McKenzie JE, Bossuyt PM, Boutron I, Hoffmann TC, Mulrow CD, et al. The PRISMA 2020 statement: an updated guideline for reporting systematic reviews. BMJ 2021;372:n71. doi: 10.1136/bmj.n71. For more information, visit: http://www.prisma-statement.org/

In case of disagreement in the screening process, the two authors discussed the inclusion/exclusion until agreement was reached. The first part of the review included 12 articles. Further, seven articles were included after the citation pearl search in Web of Science. The grey literature search resulted in two documents from the government and a health organisation, respectively, included in the scoping review. In total, 21 articles and documents were included, see the PRISMA flow chart (Moher et al., 2009) in Fig. 1. The included studies are marked with an asterisk * in the references.

### 2.4 Charting the data

Data extraction and coding stages were completed by both authors. A structured data extraction spreadsheet was created to extract data from included studies, inspired by the Cochrane Consumers and Communication Review Group's data extraction template (Ryan et al., 2016). The following information was extracted from the articles: 1) Authors, 2) Location 3) Journal, 4) Impact factor (extracted from journal website), 5) Study period, 6) Study design, 7) Sample size, 8) Target group and context, 9) Relevant results, and 10) Limitations. The extracted data were checked for accuracy by both authors. Classification discrepancies were resolved by mutual agreement. In line with Arksey and O'Mally (Arksey and O'Malley, 2005), we did not include a quality assessment of the studies. The authors nonetheless charted the studies’ strengths and limitations as reported by the respective articles’ authors. This process was first done independently, then jointly, and potential discrepancies were again resolved through in-depth discussions until a mutual agreement was reached.

### 2.5 Analytical strategy

The analysis aimed to create an overview of the knowledge in the field (Munn et al., 2018, Aromataris and Munn, 2020). The analytical phase consisted of 1) analysing the data including a descriptive numerical summary analysis and a qualitative thematic analysis, and 2) reporting the outcome articulated through themes referring to the aim of the study (Levac et al., 2010). The descriptive summary analysis was the results of the data charting, and the qualitative thematic analysis was inspired by Braun and Clark (Braun and Clarke, 2006). In the result section, the descriptive summary analysis was presented as ‘Characteristics of the studies’. The process of thematic analysis consisted of, first, the articles were read and re-read to develop a sense of the studies, facilitating familiarisation with the empirical material. Second, differences between the studies’ key findings related to the aim of the study were compared, and initial themes were constructed based on the coded material. Third, the themes were reviewed and further developed in a consensual process of analysis amongst the authors. Throughout the analysis process, the authors went back and forth between the constructed themes and the included articles to ensure that the themes appropriately reflected the content in the articles, and further that the study's aim was covered by the constructed themes. In that way, the themes and subthemes were refined, defined, and named, see Table 3. The synthesis of quantitative data and qualitative data was performed through a *qualitising* process of the quantitative data. It meant that extracted data from quantitative studies were converted into ‘textual descriptions’ to allow integration with qualitative data. The qualitising involved a narrative interpretation of the quantitative results. Within the themes, the numerical data supported or endorsed the equally important opinions and perspectives presented in interpretive and critical paradigms and vice versa (Creswell and Clark, 2017, Lizarondo et al., 2019).

**Table 3:** Themes and subthemes

Themes	Sub-themes
Medicalisation of death	The western clinical gaze and professional skills opened and closed possibilities The good death was painless and symptom-free Shared decision-making did seldom involve patients
Communication about death - a clash between two philosophies	A tension between openness and silence about death Education efforts in teaching openness about death
Dying and death were socially dependent	Death preparation as instrumental for the ‘good death’ and afterlife Family had significance for a good death and afterlife Economy at stake for the place of dying

## 3 Results

### 3.1 Characteristics of the studies

The 19 studies were carried out from 2003-2020, with data collected from 1999 to 2019. The government document found was published in 2017. The health organisation document was published in 2012 by the Chinese Associate for Life Care, which acted as a nationwide regulatory organisation in end-of-life care in mainland China. The studies were conducted in one or more places in China, located in Beijing (3), Shanghai (3), Wuhan (3), Tianjin (2), and the rest of mainland China (11). The articles were published in journals of oncology and cancer (5), palliative care and death studies (3)), ethics (4), general nursing care (4), pain and symptom management (1), and gerontology/ageing (2), respectively. The journals’ impact factor ranged from 0.918-3.783 (three journals had no impact reported)

The political documents were published at the website of the government and the Chinese Associate for Life Care. The government document was a practical guideline for palliative and hospice care. The health organisation document was a discussion paper regarding conditions for the ‘good death’ in China. A schematic overview of the included studies is presented in Table 4.

**Table 4:** Overview of the included studies

**Author(s)Study locationYear of publication**	**Journal(Year: Impact factor)**	**Aim(s) of the study**	**Study populations’ characteristics**	**Method(s)(Time period)**	**Main findings**
Dong et al. (Dong et al., 2016) Tianjin 2016	European Journal of Oncology Nursing (2019: 1.876)	To explore the experiences of Chinese physicians and nurses who provide direct care for dying patients	15 physicians and 22 nurses from different departments in a cancer centre	Face-to-face, semi-structured interview developed by phenomenology methodology (May to October 2014)	Professionals wished to provide high-quality care, including maintaining dying patients' hope. Professionals experienced ethical dilemmas when ‘hiding the diagnosis’ from patients. Professionals highly prioritised good symptom alleviation. Nurses were mostly concerned with dying patients' physical comfort and wish fulfilment, while physicians had greater emphasis on patients' rights and symptom management. Professionals encouraged relatives to express love to patients, as a well-functioning family promotes a dignified death. Professionals suffered whilst also benefiting from palliative care which helped with their personal growth and allowed greater insight into themselves and their clinical practice. Professionals call for further education related to palliative care.
Gu et al. (Gu et al., 2016) Shanghai, 2016	Supportive Care in Cancer (2019:2.635)	To investigate the factors associated with the decision making practices of Chinese terminally ill cancer patients	436 deceased patients with cancer	Retrospective data obtained from the hospital's electronic medical system (2007-2013)	Most patients (97.2 %) had signed the letter of authorisation giving family members the right to make medical decisions at end-of-life for them. Relatives felt obliged to provide aggressive or supplementary treatments in the hope of keeping the patient alive as long as possible, as giving up was considered unfilial. Half of the patients (47.3 %) received at least one of six life-sustaining treatments. Patients who lived in urban settings were more likely to undergo a life-sustaining intervention at the end-of-life period.
Pan et al. (Pan et al., 2021) South-eastern China 2021	Nursing & Health Sciences (2019: 1.269)	To describe nurses' experience of caring for terminally ill patients, and to explore how their perceptions of death and dying influence professional values To develop a theoretical framework focused on the basic social process of professional values development involved in facilitating EOL care within the Chinese cultural context	15 nurses from 3 hospitals	Qualitative interview study inspired by grounded theory as a method (2015-2016)	Three main categories were identified as ‘recognising the dilemmas when caring for terminally ill patients’, ‘applying strategies to deal with values conflict’, and ‘reconstructing values’. Nurses reported distress, helplessness, and lack of confidence and knowledge regarding the provision of end-of-life care to patients. They experienced conflict between the textbooks and clinical practice, which exposed both patients and nurses to suffering. Nurses rarely talked about death at work. Some nurses were afraid of facing death, others were committed to the dignity and physical comfort of dying patients.
Wang et al. (Wang et al., 2004) Beijing and Chinese northern provinces 2004	Journal of Pain and Symptom Management (2019: 3.077)	To explore oncology clinicians’ perceptions of barriers to providing end-of-life care	60 oncological clinicians	Survey using a self-designed questionnaire (1999)	Physicians (72%) were addressing patients’ needs including symptoms such as pain, nausea, constipation, and vomiting. Half of the physicians (51%) were asked to withhold the truth of diagnosis from patients by families, though most of the physicians (81%) found that patients should be informed. Half of the physicians (53%) made treatment decisions themselves after consulting the patient, whereas 39% found that decisions about treatments and care should be made by physicians and patients together. 48% of the physicians experienced pressures and demands from patients and relatives to continue chemotherapy beyond the medical rationale.
Yang et al. (Yang et al., 2019) Beijing 2019	European Journal of Cancer Care (2019: 2.161)	To describe the good death of terminally ill patients with cancer from the nurses' perspectives, and identify associated factors in the context of Chinese culture, to offer a new avenue for providing practical guidance to help terminally ill patients with cancer actualise a “good” death	122 nurses in charging of 258 patients	A cross‐sectional survey using Good Death Inventory and a self-designed questionnaire (2017-2018)	From nurses’ perspectives, the most important core domains for achieving a good death in cancer patients included: Being respected as an individual (89.5%); good relationship with medical staff (93.8%); and good relationships with family (83.3%). Nurses found that only few patients approached a good death in terms of independence (7.8%), physical and psychological comfort (14%), and dying in a favourite place (8.5%). Nurses found that different medical insurance coverages and payments influenced the good death of terminally ill patients with cancer.
Zheng et al. (Zheng et al., 2015) Tianjin 2015	International Journal of Nursing Studies (2019: 3.783)	To elucidate and clarify the depth of oncology nurses’ experiences of caring for the dying cancer patients who are in their final days, to enhance nurses’ end-of- life care skills and the quality of caring for the dying	28 oncological nurses	A qualitative descriptive interview study (2012-2013)	Five themes were identified: ‘End-of-life care for dying cancer patients’, ‘end-of-life care for family members’, ‘cultural sensitivity and communication’, ‘moral distress and self-limitations’, and ‘self-reflection and benefit-finding’. Nurses found that a good death included promoting fundamental physical comfort and dignity, recognising patients’ spiritual needs, and timely symptom management. Further, nurses stated the importance of the presence of family, both for patients and relatives. Often, nurses did not talk about death with patients. They were sometimes confused and felt embarrassed about deceiving patients. However, they widely accepted this practice. Nurses had a strong willingness to offer quality end-of-life care, however, they suffered moral distress because of self-limitation, lack of knowledge, confidence, and experience in palliative care.
Gu et al. (Gu et al., 2007) Random counties and cities across 22 Chinese provinces 2007	Journal of Applied Gerontology (2019: 2.144)	To explore the preferences on place of death among oldest old in China	6444 deceased persons between 80-105 years old	Retrospective data obtained from Chinese Longitudinal Healthy Longevity Survey (Conducted in 1998, 2000, and 2002).	Most of the Chinese oldest-old adults died at home (92%). Only 7% of these deceased died in hospitals. Older adults having higher socioeconomic status, being urban residents, and with family support were more likely to die in hospitals than others.
Huang et al. (Huang et al., 2015) Shanghai, Beijing, Hubei 2015	Nursing Ethics (2019: 2.597)	To explore and compare the current situation between common people and healthcare providers’ preferences for a good death in the context of Chinese culture	190 Chinese citizens and 323 healthcare professionals	Standardised questionnaire using Good Death Inventory (2009)	Participants rated that family support (96%) being respected for one's value (91%), being able to stay at one's favourite place (89%), being valued as a person (90%), free from pain (89%) and meeting people s/he wanted to see (89%) were most necessary factors in a good death. Professionals rated physical and psychological comfort, dying in a favourite place, and natural death, as more important for a good death than the public.
Huang et al. (Huang et al., 2018) Unspecified study location 2018	CambridgeQuarterly of Healthcare Ethics (2019: 1.206)	To explore the feelings and expectations of Chinese cancer patients who are going through the dying process	16 patients with advanced cancer	Mixed methods: Initial self-designed questionnaire and face-to-face interview with open-ended questions. Analysis was inspired by grounded theory and interpretive phenomenological (N/A)	Patients were aware that death was part of the normal lifespan, but rather than waiting for death, they were willing to try different oncological treatments. They wished to maintain dignity and hoped for a painless dying process with dignity and without unnecessary invasive treatments such as cardiopulmonary resuscitation. Some patients wanted information about their cancer's prognosis as this could help them accept the truth. The importance of family support and shared responsibilities amongst families were emphasised.
Ivo et al. (Ivo et al., 2012) Korea, Japan, China 2012	Journal of Medical Ethics (2019: 2.021	To understand the attitudes of cancer patients towards death and end-of-life issues and also to compare the similarities and differences across three countries	62 Chinese patients with advanced cancer	Survey using a self-designed questionnaire (2008-2009)	Most patients (80.3%) wanted to hear the truth about disease prognosis, and 54.8% of them wanted their family to be involved in decision-making processes. However, 17.7% of the patients wished to fully delegate the responsibility in decision-making medical decisions to their families. Half of the patients (55.7%) hoped to get life-sustaining treatment.
Keimig (Keimig, 2020) Kunming 2020	Anthropology Aging, (N/A)	To examine the ways end-of-life interventions complicate and compromise both living and dying for today's institutionalised elders.	Doctors, nurses, and older adults in geriatric and palliative care hospital wards and eldercare institutions	Ethnographic field study with participants observation and open-ended interviews. (2013- 2015)	Participants had an enduring need for familial connection and balance regardless of their end-of-life care setting. However, palliative care wards, nursing homes, and other eldercare settings showed that obstacles to achieve a good death, including family disharmony, economic concerns, and disease and disability, increased as dying moved out of the home and into institutions. Institutionalised older adults struggled to maintain familial and social balances necessary for achieving a ‘good death’, as social death might occur before physical death. There was a tendency towards doctors first discussing medical situations with families before revealing a diagnosis to patients. From a Chinese medicine perspective, disability, dependency, and pain could prevent the experience of the ‘good death’. Adult children of nursing home residents institutionalised their parents as a safety measure against accidental falls, sudden illnesses, and suicide.
Li et al. (Li et al., 2021) Wuhan 2021	American Journal of Hospice & Palliative Medicine (2019: 1.638)	To identify the EOL preferences among Chinese patients with cancer using the Heart-to-Heart Card Game	40 (out of 58) patients with cancer from an oncology department	Heart-to-Heart Card game consisting of 54 cards of EOL preferences were administered to patients and they were instructed to choose 3 most important ones (2019)	The three most frequently end-of-life preferences by cancer patients were: “I want my family to get along” (75%); “I don't want to be a burden to my family” (68%); and “I want to maintain my dignity” (68%); “I don't want to suffer” (65%). Maintaining a harmonious family relationship and not becoming a burden to the family were essential factors for the understanding of a good end-of-life course.
Liu van Schalkwyk (Liu and van Schalkwyk, 2019) Southern Chinese Rural city 2019	Death Studies (2019: 1.361)	To describe the elements and functions of death preparation among Chinese rural elderly	14 Chinese rural older adults	One-to-one interview with a semi-structured guideline using an adapted version of the Life Story Interview schedule. Phenomenology inspired (2016)	The older adults considered death as a part of their destiny. Talking about death was natural and they took action to prepare for death. A natural death was regarded as a good death both for one's afterlife and good for obtaining the living generations’ worship. An unnatural death could negatively affect a person and perhaps even result in him/her becoming a ‘wandering ghost in hell’. Having children, particularly sons and grandsons, was vital for getting a good death and a good afterlife. Death preparation was instrumental for a good death and afterlife. It was also a way to sacrifice oneself to save the next generations from difficulties.
Tang (Tang, 2019) Shenzhen 2019	Nursing Ethics (2019: 2.597)	To describe family caregivers’ attitudes toward death, hospice, and truth disclosure	140 primary relatives of older adults with terminal cancer at a hospice centre	Survey using a self-designed questionnaire (2017)	Two-thirds of the relatives did not encourage patients to ask about their diagnosis or to share their personal feelings about death or dying. Neither did they talk with the patient about death or dying and changed the topic when talk about death was discussed. From the perspectives of relatives, older adults with terminal cancer who knew their diagnosis were more likely to have a positive attitude toward death, have family around them, and die at home, whereas those unaware of their diagnosis were more likely to refuse to discuss death with their relatives and to feel more distressed.
Zhang (Zhang et al., 2013) Quzhou 2013	Journal of Nursing (China) (N/A)	To explore family members' perception and expectation about the good death among cancer patients’ family members	12 relatives of patients with cancer	Face-to-face Interview with open-ended questions (January-July,2012)	Three dimensions of a good death were identified by family relatives: Control of patients’ physical symptoms, such as providing pain-relief intervention; meeting the expectations of patients' psychological needs; and fulfilling patients’ expectations and needs from a societal aspect such as having support from medical staff. Relatives found that the support from nurses, family core members, and other patients in the ward helped them to accept the death of their beloved.
Liu et al. (Liu et al., 2016) Sichuan 2016	Chinese Nursing Research (N/A)	To investigate the factors that influence family members' EOL decision-making process	10 relatives of patients in end-of-life intensive care	Semi-structured in-depth interview based on phenomenology methodology (N/A)	Three influencing factors of clinical decision of family members of end-of-life patients in ICU was seen: Objective factors such as quality of life, prognosis of the disease, lifetime wishes, and death place; family factors such as economic condition, and whether relatives’ views unified or not; and social and cultural factors such as filial piety, fallen leaves return to the roots, and death attitude. Shaped by the Chinese ‘filial piety’ philosophy, relatives wanted to continue treatment even if patients had a poor prognosis as this was a way to repay patients. From the perspective of relatives, allowing patients to die at their own place was the best way to repay them. Factors such as poor quality of life, poor prognosis, patients’ own wishes, and family financial status were major factors that lead relatives to make a ‘giving up’ decision.
Chen et al. (Chen et al., 2020) Wuhan 2020	Asian Nursing Research (2019: 0.988)	To investigate the effectiveness of a structured death education program	80 older adults with chronic illness and 80 related relatives	Two-group, non-randomised quasi-experimental study consisting of 5 weeks bedside death education was delivered to the intervention group and 5 weeks usually health education to the placebo group (July-December 2019)	Death education improved patients' death attitudes and satisfaction in relation to ‘usual practice’ (p=.002). Patients’ fear of death was significantly reduced by the death education program (p < .001). Death education programs promoted the transition of the patients’ the patients’ death avoidance to neutral acceptance.
Cui et al. (Cui et al., 2011) Shanghai 2011	Oncology Nursing Forum (2019: 1.728)	To identify the components that registered nurses needed most about death education	617 registered nurses working in seven hospitals	A cross-sectional survey using a self-designed questionnaire (N/A))	Nurses reported the highest score (mean=4.13, SD=0.66) on managing death and dying issues in death education. The death education could help nurses understand death and dying philosophy, supporting them in handling their own psychological stress. Nurses regarded the good death as good communication with patients and their families about death and dying, where they were able to handle ethical issues. Nurses need education regarding coping skills and knowledge about death and dying when they meet different situations.
Deng et al. (Deng et al., 2014) Wuhan 2014	American Journal of Hospice and Palliative Medicine (2019: 1.638)	To evaluate the efficacy and safety of diverse analgesic adjustment strategies among hospice patients with uncontrolled pain	728 hospice patients with advanced cancer and uncontrolled pain from a hospice centre	Retrospective cross-sectional study with data retrieved from the medical records system from 2003 to 2010 in the hospice centre (N/A)	No significant differences were found between different analgesic efficacies and clinical characteristics. Physicians had difficulties identifying the pain's causes, which resulted in difficulties in selecting appropriate pain-free analgesics.
National Health Commission of People's Republic China (National Health Commision of People's Republic China 2017) 2017	National Health Commission of People's Republic China. http://www.nhc.gov.cn/cms-search/xxgk/getManuscriptXxgk.htm?id=3ec857f8c4a244e69b233ce2f5f270b3	To serve as a guideline for establishing a palliative and nursing care framework	-	Political document: Memorandum of Practice Guidelines for Palliative Care and Nursing (for Trial Implementation)	Good palliative care aimed at managing patients’ symptoms effectively and at ensuring the patients’ quality of life, which could be handled through overall symptom control (including pain control followed by WHO Analgesic ladder strategy, difficulty breathing, coughing, coughing out blood, nausea, etc.); providing comfortable care; and patients’ psychological, spiritual, and social support. A comfortable living environment and specialised palliative nursing care (such as oral care nutritional care) should be provided for palliative patients. Psychological support was aiming at establishing trusting relationships between medical staff and patients, to guide patients to accept the prognosis and cope with emotional reactions, encourage patients and families to participate in decision-making processes, and respect patients’ wishes.
Chinese Association for Life Care (Chinese Association For Life Care 2012) 2012	Homepage for the Chinese Association for Life Care. http://www.cnaflc.org/zxzx/6548.jhtml and https://hospicecare.com/global-directory-of-providers-organizations/listings/details/733/	To briefly describe the challenges that Chinese palliative care system is facing	-	Organisational document: Palliative care: A challenge to achieve the ‘good death’	The document briefly argued why western inspired palliative care is needed in China, and further, it discussed the challenges the Chinese palliative care system must face.

The research aim of six studies was to explore Chinese healthcare professionals’ experience of and/or attitudes to caring for dying and death (Dong et al., 2016, Gu et al., 2016, Pan et al., 2021, Wang et al., 2004, Yang et al., 2019, Zheng et al., 2015). Nine studies focused on understandings of death and dying from patients’ and relatives’ perspectives (Gu et al., 2007, Huang et al., 2015, Huang et al., 2018, Ivo et al., 2012, Keimig, 2020, Li et al., 2021, Liu and van Schalkwyk, 2019, Tang, 2019, Zhang et al., 2013). Two studies explored the components of the ‘good death’ by focusing on end-of-life decision-making processes (Gu et al., 2016, Liu et al., 2016). Two studies had primarily educational aims (Chen et al., 2020, Cui et al., 2011), and one about pain management (Deng et al., 2014). However, the ‘good death’ was touched in these studies.

Seven studies used qualitative research designs, whereas six studies used semi-structured interviews (Dong et al., 2016, Pan et al., 2021, Zheng et al., 2015, Liu and van Schalkwyk, 2019, Zhang et al., 2013, Liu et al., 2016) and one was an ethnographic field study, using both observations and semi-structured interviews (Keimig, 2020). Eleven studies used quantitative designs, whereas two studies were based on standardised questionnaires (Yang et al., 2019, Huang et al., 2015), five used self-designed questionnaires (Wang et al., 2004, Gu et al., 2007, Ivo et al., 2012, Tang, 2019, Cui et al., 2011), two used empirical material from medical records (Gu et al., 2016, Deng et al., 2014), one used a non-randomised controlled design (Chen et al., 2020), and one used selected game cards (Li et al., 2021)**.** One study used mixed methods, combining a self-designed questionnaire and semi-structured interviews (Huang et al., 2018).

The exact numbers of the study population were unknown, as the anthropological field study (Keimig, 2020) included an undefined group of actors in the studied field. The other 18 studies represented 9392 participants. It included 1202 registered nurses and physicians where the majority worked at hospitals, 958 patients with different chronic diseases, including cancer, 6880 deceased persons, 242 relatives to terminally ill patients, and 190 citizens from the public. The participants’ ages ranged from 18 to 105, despite in one study including participants from 16+ years old (Huang et al., 2015). Most studies did not refer to any theories. However, two of the qualitative studies had a theoretical methodical framework (Keimig, 2020, Liu and van Schalkwyk, 2019), but the theories did not drive the result analysis nor the discussions. Four studies had a defined concept of the ‘good death’ (Yang et al., 2019, Huang et al., 2015, Keimig, 2020, Zhang et al., 2013), while 15 studies had an implicitly description of the ‘good death’ through use of related factors such as ‘pain control’ (Deng et al., 2014), ‘attitude to death’ (Dong et al., 2016, Pan et al., 2021, Wang et al., 2004, Zheng et al., 2015, Huang et al., 2018, Ivo et al., 2012, Tang, 2019), ‘place of death’ (Gu et al., 2007)’, ‘end-of-life preference’ (Li et al., 2021), ‘death preparation’ (Liu and van Schalkwyk, 2019), ‘death education’ (Chen et al., 2020, Cui et al., 2011), and ‘decision-making process’ (Gu et al., 2016, Liu et al., 2016).

Most of the quantitative studies presented well-defined outcome measurements and the reasoning of statistical analysis methods. Two studies using self-designed survey instruments had a detailed description of their survey instruments (Wang et al., 2004, Cui et al., 2011), and three studies had no description of the development of their instruments (Huang et al., 2018, Ivo et al., 2012, Tang, 2019). None of these studies attached the questionnaire. The studies using standardised instruments in a Chinese version (Yang et al., 2019, Huang et al., 2015) were validated and tested according to the international standard for translation of measurement tools. Both the intervention and the control arms in the non-randomised quasi-experimental study generally lacked description. Only the headings of the content in the intervention were mentioned, and the control arm was described as ‘usually’ education/care, with unknown content to the readers (Chen et al., 2020). One retrospective study included a large sample from nationwide surveys but lacked the information of questions asked in the survey (Gu et al., 2007). Most of the qualitative studies described the interview questions in a brief manner, but only one study included the interview guide (Huang et al., 2018). All qualitative studies described the analytical strategies, except one study (Keimig, 2020). There was generally a lack of reflection about the study's methods and brief comments on the limitations. Five studies did not report any limitations (Huang et al., 2018, Keimig, 2020, Zhang et al., 2013, Liu et al., 2016, Cui et al., 2011). All other studies reported a varied number of limitations. Eight studies reported limitations relating to the samples’ characteristics, e.g., diagnosis, socio-economic status, sample size, and generalisability (Dong et al., 2016, Pan et al., 2021, Yang et al., 2019, Zheng et al., 2015, Huang et al., 2015, Ivo et al., 2012, Liu and van Schalkwyk, 2019, Chen et al., 2020). Moreover, studies reported methods limitations such as using retrospective data and recruitment strategy (Liu and van Schalkwyk, 2019, Deng et al., 2014), single study context (Gu et al., 2016), and study contexts with only specialised healthcare professionals (Pan et al., 2021, Wang et al., 2004). Studies reported risk of bias related to the objectivity and construction of self-designed/standardised measurement instruments (Yang et al., 2019, Gu et al., 2007, Huang et al., 2015, Tang, 2019, Chen et al., 2020). Two qualitative studies reported limitations relating to gender bias (Dong et al., 2016, Zheng et al., 2015).

### 3.2 Medicalisation of death

#### 3.2.1 The western clinical gaze and professional skills opened and closed possibilities

The western clinical gaze, understood as the medical observations, diagnostic and treatment perspective pertinent to a person's care, formed the understanding of the ‘good death’, where it seemed as if any treatment was better than no treatment. Studies showed that nurses focused often on the dying patients' physical comfort, including cleaning and dressing, turning over in bed, mouth care, and wish fulfilment (Dong et al., 2016, Zheng et al., 2015). The physicians placed great emphasis on patients' rights and symptom management (Dong et al., 2016, Wang et al., 2004). The physicians rated their symptom management skills as very competent for physical symptoms for patients in their last six months of life but poor or fair competencies for managing symptoms of psychological character such as anorexia and depression. Further, 80% of the physicians would continue giving anti-cancer agents up to the end of life, whereas 20% would stop if a patient had stage III–IV (Wang et al., 2004). Physicians and nurses supported the medical importance and urgency of symptom management for dying cancer patients, which was regarded as an important element in the understanding of the ‘good death’ (Dong et al., 2016, Zheng et al., 2015). The document by the National Health Commission of People's Republic China supported the importance of the western clinical gaze and medical logic in palliative care (National Health Commision of People's Republic China 2017). It highlighted the provision of specialised nursing care and overall symptom control in palliative care included pain control, difficulty breathing, coughing, and nausea to ensure patients’ quality of end of life (National Health Commision of People's Republic China 2017). The Chinese Association for Life Care defined palliative care as a kind of advanced health and medical service, aiming at supporting the dying patients and their relatives arguing that western inspired organised palliative care was important to facilitate the ‘good death’ in China (Chinese Association For Life Care 2012). However, nurses meant that providing intensive interventions should consider patients’ health condition, and they found fundamental care, spiritual support, and maintenance of dignity as important elements to create the 'good death' (Zheng et al., 2015). The Chinese Practice Guidelines for Palliative Care and Nursing also pointed out that patients should be respected individually providing psychosocial support for a peaceful end-of-life (National Health Commision of People's Republic China 2017). Dignity was mentioned as an aspect of the ‘good death’ by nurses, physicians, patients, and relatives (Dong et al., 2016, Pan et al., 2021, Zheng et al., 2015, Huang et al., 2018, Li et al., 2021, Zhang et al., 2013). Nevertheless, all actors had different perceptions of how to achieve dignity in end-of-life. Nurses and physicians attempted to maintain patients’ dignity by fulfilling dying patients' wishes (Dong et al., 2016, Zheng et al., 2015, Zhang et al., 2013), facilitating death in place, and being gentle to unconscious patients (Dong et al., 2016). Nurses also stated that prolonging incurable diseases and dying patients’ life would disregard patients’ dignity (Pan et al., 2021, Zheng et al., 2015). There were crevices in the clinical diagnostic and treating gaze, where co-humanity also was found in the physicians' approach to patients such as holding patients’ hands or shaking hands to maintain patients’ hope for living (Dong et al., 2016). Dignity was also perceived as an important preference at end-of-life among patients with cancer (Li et al., 2021). In Huang et al.’s study, 93% of the patients did not wish to receive any invasive treatment or cardiopulmonary resuscitation to keep their dignity (Huang et al., 2018), while Ivo et al. showed that 55.7% of the cancer patients supported life-sustaining treatments (Ivo et al., 2012). Gu et al. revealed that 40% of the patients received parenteral nutrition and hydration to avoid 'starving to death', as this was considered an unacceptable death in China (Gu et al., 2007). Zhang and colleagues showed that some relatives insisted on stopping invasive treatment such as tracheostomy, while other relatives wanted to prolong the patient's life for any price, where both choices were justified in patients’ dignity (Zhang et al., 2013).

Studies showed that the ‘good death’ could be measured through a variety of assessments (Yang et al., 2019, Huang et al., 2015, Chen et al., 2020). The studies acknowledged several qualitative concepts of importance for experiences and understandings of the 'good death'. In Chen et al.’s study, death attitude was measured by the Chinese version of Death Attitude Profile-Revised (DAP-R), Death competence was measured using Bugen's Coping with Death Scale (CDS), and Well-being Index (WHO-5) (Chinese language version) was used to assess patient well-being (Chen et al., 2020). The family function was evaluated using the Family APGAR, and Patient satisfaction was assessed by the Client Satisfaction Tool (CST) (Chen et al., 2020). Good Death Inventory was used in two studies to measure important elements of the ‘good death’ from perspectives of the Chinese public and nurses (Yang et al., 2019, Huang et al., 2015). The underlying idea seemed to be that relevant issues for the ‘good death’ could be measured relative simply by putting a cross on a scale and counting the numbers.

### 3.2.2 The good death was painless and symptom-free

Symptom and pain-control interventions were reported as an imperative factor to ensure patients the ‘good death’ from perspectives of all actors (Dong et al., 2016, Wang et al., 2004, Huang et al., 2015, Li et al., 2021, Zhang et al., 2013, Deng et al., 2014). Patients expressed that the ideal ‘good death’ was dying without pain and physical sufferings (Huang et al., 2018, Li et al., 2021, Zhang et al., 2013). Dong et al. showed that both physicians and nurses considered pain and physical symptom control as the top priority to achieve quality of death (Dong et al., 2016). Deng and colleagues emphasised the importance of effective analgesic strategies for patients to avoid uncontrolled pain (Deng et al., 2014). Another study found that two-thirds of the physicians reported being highly competent in managing cancer pain, including standardised pain assessments and side-effect management (Wang et al., 2004). The government document highlighted that pain control in palliative care should follow WHO Analgesic ladder strategy, selecting the appropriate pain assessment tool according to the patient's cognitive ability (National Health Commision of People's Republic China 2017). The Chinese Association for Life Care argued that relieving pain for dying was the best way to preserve patients’ dignity (Chinese Association For Life Care 2012). In the study of Huang et al, ‘physical and psychological comfort’ was mentioned as one of core elements of a good death by 80% participants from the Chinese public, including healthcare professionals (Huang et al., 2015). Death brought on by accidents, failed surgery, or suicide was interpreted as a painless and symptom-free death, which, according to Liu and van Schalkwyk, was significant for an individual's afterlife and thus important for obtaining the living generations’ worship (Liu and van Schalkwyk, 2019). A painful death could negatively affect individuals and even result in their becoming wandering ghosts in hell (Liu and van Schalkwyk, 2019).

### 3.2.3 Shared decision-making did seldom involve patients

Encouraging patients' involvement in decision-making had been advocated by the National Health Commission of People's Republic China. The commission argued that these practices ensured respect for patients’ wishes and were necessary for patients to achieve optimistic attitudes during end-of-life (National Health Commision of People's Republic China 2017). However, many patients preferred a passive-role in the decision-making process, where relatives were assigned active roles in medical treatment decisions on behalf of patients (Gu et al., 2016, Huang et al., 2015, Ivo et al., 2012). The decisions of end-of-life issues were influenced by filial piety, which in a Chinese context was understood as obeying and taking care of an ill person. It included being tacit about diseases and imminent death as it had significance for the individual's passing and afterlife (Liu et al., 2016, Chinese Association For Life Care 2012). Liu et al. reported that some relatives had refrained from further treatment on behalf of the patients due to poor quality of life or poor prognosis. Patients were convinced that relatives had intentions to decide the best way to minimise patients’ suffering (Liu et al., 2016). Conversely, other studies showed that relatives often felt obliged to support aggressive or supplementary treatments in the hope of keeping the patient alive at the longest possible (Gu et al., 2016, Wang et al., 2004). Wang et al showed that 48% of the physicians experienced a heavy demand from patients and relatives for continuing chemotherapy (Wang et al., 2004). In addition, studies reported that physicians often chose to communicate the diagnosis with relatives before they consulted patients (Dong et al., 2016, Wang et al., 2004, Keimig, 2020). Further, 53% of the physicians made treatment decisions themselves after consulting the patient, where 39% found that decisions about treatments and care should be made by physicians and patients jointly (Wang et al., 2004). Depression was the major concern why 66% of the physicians would only tell the ‘truth’ if patients requested it (Wang et al., 2004). Zheng and colleagues pointed out the importance of keeping dying patients from the information of imminent death for the sake of maintaining patients’ hope, beliefs, and confidence in recovering (Zheng et al., 2015). However, from nurses’ perspective, relatives should tell dying patients the truth because patients should know their days, so they have the possibilities to prepare or fulfil their wishes for life and death (Zheng et al., 2015).

### 3.3 Communication about death - a clash between two philosophies

#### 3.3.1 A tension between openness and silence about death

According to the National Health Commission of People's Republic China, healthcare professionals should guide patients and relatives to accept the diagnosis and help them in coping with their psychological stress (National Health Commision of People's Republic China 2017). Communication about death at end-of-life was often reported as challenging for all actors involved. Several studies indicated that healthcare professionals hardly touched death with dying patients (Dong et al., 2016, Pan et al., 2021, Zheng et al., 2015, Cui et al., 2011), as talking about death was a taboo (Zheng et al., 2015), seen as a ‘bad luck’ in the afterlife for the patients (Dong et al., 2016). It might also recall nurses’ personal feelings such as grief and anxiety (Cui et al., 2011). The perception of death was related to life philosophies such as Confucianism and Taoism and was also connected to Chinese ghost stories (Pan et al., 2021). Some nurses believed the patient's spirit remained after they had died, so nurses would not look at patients' faces during post-mortem care or were afraid of recalling the scenes of patients' death. Within this cultural context, healthcare professionals developed a certain approach of communicating death with patients at terminal stages. For example, Dong and colleagues (Dong et al., 2016) showed that physicians and nurses used the word ‘the disease’ instead of the words ‘cancer/tumour’ when communicating with patients, which also was a strategy to maintain the patient's hope for cure (Dong et al., 2016, Zheng et al., 2015). Nonetheless, healthcare professionals expressed dilemmas at work and emotional sufferings during end-of-life care. Physicians and nurses questioned this ‘hiding-diagnosis-from-patients’-practice (Dong et al., 2016). Nurses expressed difficulties to maintain the false hope to patients (Dong et al., 2016) and were confused about deceiving dying patients from their real health conditions (Zheng et al., 2015). A gap was seen between palliative care textbooks guiding practice in direction of a normative ideal and clinical practices sparingly reflecting dying patients’ situations and needs (Pan et al., 2021). Physicians and nurses indicated that patients should be informed about their conditions (Dong et al., 2016, Wang et al., 2004, Zheng et al., 2015). However, Wang et al reported that 51% of the physicians were asked by relatives to withhold the ‘truth’ from patients (Wang et al., 2004). Relatives hid diagnosis from patients to shield patients from distress and worries (Dong et al., 2016, Tang, 2019). Neither did relatives talk about death and dying with patients, nor did they encourage patients to share feelings of death (Tang, 2019). Conversely, Ivo and colleagues showed that most patients with advanced cancer wanted to be informed about their diagnosis (Ivo et al., 2012). Patients who knew their diagnosis were more likely to develop positive attitudes towards death, whereas patients who did not know about their diagnosis were more likely to refuse to talk about death, and their relatives were more likely exposed to high psychological burdens (Tang, 2019). Studies showed that older adults living in rural areas were comfortable telling near-death experiences, their impending or other's death in community (Huang et al., 2015, Liu and van Schalkwyk, 2019).

### 3.3.2 Education efforts in teaching openness about death

The importance of death education was highlighted by both patients, relatives, and professionals. Death education could be seen as an indirect element of the ‘good death' as it was an approach to enhance understanding and acquired knowledge of death and palliative care among professionals, patients, and relatives. Chen and colleagues showed that patients who received weekly bedside death education had reduced fear of death and improved quality of life compared to those patients who received ‘usually’ health education (Chen et al., 2020). A study showed that a death education programme allowed nurses to understand death (care) and helped nurses to deal with their own psychological stress, which was indirectly understood to also indulge in providing better end-of-life care to patients (Cui et al., 2011). However, studies also showed that there was a lack in death education and training in hospitals (Dong et al., 2016, Pan et al., 2021, Zheng et al., 2015, Cui et al., 2011). In addition, physicians reported lack of information in the literature for communicating death and related issues with patients (Dong et al., 2016)**.** Also, the Chinese Association for Life Care argued for palliative care education of healthcare professionals to make optimal conditions for framing the ‘good death’, however, only a few educational institutions in China have launched death education and bioethics education courses (Chinese Association For Life Care 2012).

### 3.4 Dying and death were socially dependent

#### 3.4.1 Death preparation as instrumental for the ‘good death’ and afterlife

Death preparation was regarded as both a practical and a mental issue of importance for the ‘good’ life ending, the ‘good death’, and the ‘good afterlife’. Liu and van Schalkwyk found that the ‘good death’ was contextualised in the informants’ rural surroundings, where conversation about death and death preparation were common practices (Liu and van Schalkwyk, 2019). Death preparation was regarded as a condition for the ‘good death’ and afterlife, and a way to sacrifice individuals to save the next generations from difficulties. Many of the older adults from the rural settings acted when they still were healthy, e.g., decisions about coffin and burial clothing as this were associated with the ‘good death’. Zheng and colleagues showed that nurses sometimes found relatives neither aware of the dying process, nor did they know how to prepare themselves for the patients’ death (Zheng et al., 2015). Some nurses helped relatives be emotionally prepared for the patient's impending death without losing hope and faith, while other nurses hardly talked about death and dying with the patients due to Chinese traditional culture (Zheng et al., 2015).

#### 3.4.2 Family had significance for a good death and afterlife

Several studies stressed the significance of good familial bonds for a good dying process and the ‘good death’ (Yang et al., 2019, Huang et al., 2015, Keimig, 2020, Li et al., 2021, Liu and van Schalkwyk, 2019, Liu et al., 2016). Keimig showed family disharmonies were obstacles to achieving a ‘good death’, and older adults in institutions struggled to maintain the familial and social balances necessary for the ‘good death’ (Keimig, 2020). Studies argued that even disability, dependency, pain, and death affected the proper balance according to principles of Chinese medicine (Keimig, 2020, Li et al., 2021). Social death occurred long before physical death releases for some older adults in institutions (Keimig, 2020). In that way, disharmonies in families prevented the experience of the ‘good death’. Huang et al. also showed the importance of relatives where the components that received the highest mean scores regarding the ‘good death’ among Chinese citizens were having family support, being able to stay at one's favourite place, being valued as a person, and meeting people they wanted to see (Huang et al., 2015). According to Liu & van Schalkwyk, having sons and grandsons were essential for older adults for achieving the ‘good death’ and afterlife in rural contexts, while daughters were treated as outsiders of the family once, they got married (Liu and van Schalkwyk, 2019). Older adults believed that the ‘good death’ meant children gathering around the dying bed, but the oldest son should be the one performing funeral rituals and ceremonies (Liu and van Schalkwyk, 2019).

The understanding of the ‘good death’ was also a question not overburdening relatives, both before and after death (Huang et al., 2018, Keimig, 2020, Li et al., 2021, Liu and van Schalkwyk, 2019). Keimig showed that by preparing their own death-related rituals, individuals could lessen their children's burdens at the time of their death (Keimig, 2020). Liu & van Schalkwyk (Liu and van Schalkwyk, 2019) found that the ‘good death’ could be suicide in some Chinese rural areas, as suicide was seen as a planned action of sacrifice heroically for others good. However, some relatives did not find suicide as the ‘good death’ (Keimig, 2020). Relatives to nursing home residents made their decision to institutionalise their parents to safeguard the parents, not only from accidental falls and sudden illnesses, but also from committing suicide. Some patients pointed to the possibility of euthanasia (Huang et al., 2018).

Further, studies showed that relatives functioned as assistants to professionals in the medical field, helping and supporting the patients in the terminal phase of their lives. Physicians and nurses stated the importance for patients and relatives of having relatives being together with the dying person (Dong et al., 2016, Zheng et al., 2015). Nurses believed that relatives should not leave the patients alone, using positive physical contacts like holding hands, touching, or having eye contact, which were understood as meaningful ways of expressing their love (Dong et al., 2016, Zheng et al., 2015). Nurses also supported relatives by providing certain caring skills in caring for dying persons (Dong et al., 2016).

### 3.4.3 Economy at stake for the place of dying

According to the National Health Commission of People's Republic China, it was important to provide a comfortable living environment for dying patients (National Health Commision of People's Republic China 2017). Dying at one's favourable place was a privilege, which was closely linked to the persons' socio-economic status. The place for lived life had also an impact on where death could take place. Gu and colleagues showed that individual sociodemographic characteristics, health conditions, and health resources affected the place of death (Gu et al., 2007). Individuals from relatively developed communities, with relatively higher socioeconomic status, and individuals having pension and/or free medical services in mainland China tended to have a higher chance of hospital and/or institutional deaths than others (Gu et al., 2007). Dying in hospitals or other reputable medical clinics was perceived as the good place to die, with the right conditions to be able to get the ‘good death’. Keimig showed that older adults who were disabled or care-dependent were left in economic ruin and had to choose poorly regulated caregiving facilities in which they spend their lives until death (Keimig, 2020). This meant that the ‘good death’ was related to and a matter of social class in Chinese society. From the perspective of relatives, dying at one's own place was the best way to repay the dying person, however, it was also a matter of economy (Liu et al., 2016).

## 4 Discussion

This discussion focuses on three main findings. First, we discuss how the western clinical gaze also has found its way into palliative care in the Chinese health system, and how the ‘good death’ is understood in the tension of those two logics. Second, we discuss how there is a clash between the western inspired idea about openness about death and dying and the traditional Chinese silence around death and dying. Third, we discuss relatives’ significance for the ‘good death’ in mainland China, and the implication for the way they are included and excluded in the medical palliative field. Finally, we discuss the strengths and limitations of the current scoping review.

The results showed that good symptom control and pain management were emphasised as a key component in achieving the ‘good death’ by all actors in mainland China, which is also shown in other studies and in line with the overall western philosophy of palliative care (Meier et al., 2016, Miyashita et al., 2007). The use of measuring tools in current study points to an instrumentalisation and a medicalisation of everyday life in the process of dying. The use of measurement tools reduced qualitative concepts to a simplicity that could be measured with a standardised questionnaire and translated into and expressed through the appearance of a number. A similar pattern is seen in the use of measurement tools in reablement practice development projects, where an instrumentalisation and a medicalisation of activities in everyday life were seen (Glasdam and Sudmann, 2021). Measuring tools tend to produce generalised truths about individuals and were used to predict outcome of or access to reablement programs, guiding both patients and professionals (Glasdam and Sudmann, 2021). As social technologies, the use of measuring tools is powerful in producing generalised truths about death and dying, without taking the subjective and qualitative aspects of death and dying into consideration. Use of measuring tools had an intrinsic power to transform natural movement in everyday life into a logic of western medicine with a need for lifestyle change, serving to maintain the vital functions of the body (Nicholls et al., 2018, Pedersen and Saltin, 2015), even in the end-of-life. This contrasts with the Chinese philosophy of death highlighting harmony, understood as both an inner harmony and being in harmony with relatives (Hsu et al., 2009). Western medical individualism has a significant impact on healthcare in China (Bian, 2015). Traditional Chinese ethical culture has long influenced the consciousness, spirit, ideas, and moral practices of the Chinese people, which are embedded in practices of families and society. The Chinese way of living is essentially family based and family oriented (Bian, 2015), although such cultural elements have undergone significant changes under the capitalist economy and China's open-door policy since the 1970s with increasing focus on individual achievement and individualism (Kolstad and Gjesvik, 2014). The current study showed that practices and visions for palliative care in mainland China are influenced by western individualistic values. As a result, fundamental and moral tensions are seen between the healthcare practices operating within both China's familistic culture and the western medical logic of palliative care. Hong Kong might be a benchmark for what movement is underway. Through many years, the healthcare system in Hong Kong is based on the United Kingdom National Health Service (Lu et al., 2018), and the development of palliative care in Hong Kong is supported by legislation and collaboration among various clinical specialties within individual-based medicine (Cheng et al., 2019). Although the adoption of individual-based medicine was implemented into the local legislative framework, studies show that familistic decision-making processes are preferred (Chan et al., 2015, Cheung et al., 2020). This illustrates how historically incorporated patterns and habits take generations to change (Bourdieu, 2016), why it is not possible to change basic human structures, built up over many generations, only by political and medical decisions.

Further, the results showed that there was a clash between the western inspired idea about openness about death and dying and the traditional Chinese silence around death and dying. The Chinese philosophy of death and dying were influenced by a mixture of Confucianism, Taoism and Buddhism (Hsu et al., 2009). Taoism regards death as a natural process of life and as an inauspicious event that should be avoided, and physical life should be prolonged. Confucianism regards death as an inevitable event in life, emphasising that people should not talk about death and/or afterlife as a respect to ghosts and spirits, but concentrate on individual development (Hsu et al., 2009). Buddhism regards death as an inescapable fact of life and the sooner individuals understand the condition, the better they could take necessary actions. Further, Buddhism fills the gap of ancient Chinese philosophy regarding afterlife (Guang, 2013). In contrast to the western culture focusing on open communication and patient's independence and autonomy, contemporary Chinese medical practice remains silent in terms of truth-telling of prognosis and diagnosis to patients (Fan and Li, 2004). The current results showed that talking openly about death was seen as taboo and socially inappropriate among several individuals in mainland China, however, there was also a shift from avoiding death disclosure towards wishing to have open dialogues about death and dying. This occurring transformation is partly influenced by contemporary western medicine practice (Nie, 2011).

Moreover, the results showed that relatives were placed in a tension between the medical logic and the traditional Chinese values. They partly were expected to be assistants to the professionals supporting the medical logic of the ‘good death’. And, partly, they were important for the harmony in the dying person's life, which was regarded as an element in the ‘good death’ and afterlife. However, patients did not want to burden relatives, which also is shown in other studies (Kim, 2019, Miyashita et al., 2007. In the western clinical practice, professional support to relatives is rarely included in the way that treatment and care are organised in healthcare (Glasdam and Oute, 2019). Professionals regard relatives within a medical-professional rationality in which relatives are not regarded as a subject of care and support in clinical practice, but they aim to help professionals to care for patients when the professionals were not absolutely needed (Glasdam and Oute, 2019). Other studies show how there is a gap between the western philosophy of palliative care and the clinical practice of palliative healthcare, where the western medicine logic of diagnostics and treatment overrules the philosophy of palliative care, also in western countries (Graven and Timm, 2019, Glasdam et al., 2020, Karidar et al., 2016, Karidar and Glasdam, 2018). Taking care of a patient is usually an obligation for Chinese families (Fan and Li, 2004), and the place of dying is as significant for Chinese families as also found in other countries (Meier et al., 2016, Miyashita et al., 2007). Studies show that family relatives’ willingness to learn from nurses about how to take better care of patients in mainland China (Cheng and Chen, 2021, Cui et al., 2014). However, the relatives are often distinct from necessary knowledge about caring for their beloved ones and usually lacking support from healthcare professionals (Cheng and Chen, 2021, Cui et al., 2014).

The current scoping review has both strengths and limitations. Scoping reviews are especially relevant for mapping research areas with emerging evidence and a lack of randomised controlled trials such as studies about understandings of death and dying in China, as studies with a range of study designs can be included in the mapping (Levac et al., 2010). Including grey literature in the review made it possible also to highlight the authority perspective on the ‘good death’ in mainland China as this perspective was not found in the research literature. The search resulted only in 117 identified studies, which was why the construction of search strings was discussed with a university librarian to rule out that no significant elements in the search had been overlooked. It could maybe have strengthened the search strategies if the database Embase® also was used. In addition, the grey literature search could have been strengthened by engaging the government and health authorities in the strategies. However, the strategy chosen was the one that came closest to finding articles that corresponded to the aim of the study. In other words, there is a limited number of studies in this area and the chosen search strategy seems to capture them. Further, it can be discussed if it is a limitation not doing quality assessments in the scoping review as the quality of the results’ evidence is unknown*.* However, in line with Levac et al. (Levac et al., 2010), we recognise challenges in assessing quality in the grey literature included in the scoping review, for example how should a discussion paper be quality assessed? It does not clarify the evidence of the results that some of the included literature is quality assessed and other is not. Moreover, some of the included studies had not a primary focus on ‘good death’. However, it was possible to analyse the understanding of the ‘good death’ indirectly through their focus on for example 'quality of death' and ' attitudes to death'. This can be regarded as both a strength and a limitation of the study as the result presents a broad range of perspective on the ‘good death’, and, however, there is a risk of mis- or overinterpretation of the articles. The included studies are mainly of individuals living in urban cities, who have better health services and different medical insurance coverage than in rural areas in mainland China, which may affect the transferability of current results outside the contexts of the included studies.

## 5 Conclusion

The study showed that the understanding of the ‘good death’ in mainland China was a negotiation between Chinese traditional philosophy and contemporary western medicine practice. There seemed to be a clash between the two cultures in the understanding of a good death, where western philosophy seemed to rule the authority and medical actors while traditional Chinese logic seemed to rule parts of the population. In mainland China, palliative care and death often took place in institutions, where the understanding of the ‘good death’ primarily focused on physical symptoms and treatments. A variety of tools were used to measure the outcome of a good death, supporting a medicalisation of and simplification of the understanding of the good death. The influence of western philosophy of palliative care advocated openness about death, trapping inhabitants, including healthcare professionals, in mainland China between traditional values and modern western medical perspectives of a good death. In the traditional understanding of the ‘good death, talking openly about death was seen as taboo and socially inappropriate. Relatives could support or prevent good conditions for the dying person, which was regarded as significant for the ‘good death’ and a good afterlife in mainland China. The understanding of the ‘good death’ consisted partly of a timely and practical preparation for the death, passing and afterlife, partly it was a matter of social and financial issues. The ‘good death’ was also regarded as a matter of healthcare professionals' competencies and education. This study calls for further research about palliative care, including the ‘good death’, where relational perspectives in the processes and practices of palliative care and dying might be explored to understand the complexity of the influence of western philosophy and traditional Chinese philosophy for both dying persons, relatives, and healthcare professionals. Additionally, further research is needed to explore the ‘good death’ within diverse geographical and social contexts. Moreover, the gap between palliative care textbooks guiding practice in direction of a normative ideal and clinical practices sparingly reflecting dying patients' situations and needs calls for future research.

## Funding sources

No funding to declare

## Declarations

• Data availability statement: public available
• No funding to declare
• No conflict of interest
• The study was carried out in accordance with ethical research principles as stated in the Declaration of Helsinki (World Medical Association 2013).

## Author Contribution

**Cong Fu**: Conceptualisation, Methodology, Investigation, Writing- Original draft preparation, Writing - Review & Editing., **Stinne Glasdam**: Conceptualisation, Methodology, Investigation, Writing- Original draft preparation, Writing - Review & Editing

## Declaration of Conflict of interest

There are no conflict of interest
